# Washeteria closures, infectious disease and community health in rural Alaska: a review of clinical data in Kivalina, Alaska

**DOI:** 10.3402/ijch.v72i0.21233

**Published:** 2013-08-05

**Authors:** Timothy K. Thomas, Jake Bell, Dana Bruden, Millie Hawley, Michael Brubaker

**Affiliations:** 1 Division of Community Health Services, Alaska Native Tribal Health Consortium (ANTHC), Anchorage, AK, USA; 2 Centers for Disease Control and Prevention/Arctic Investigations Program (CDC/AIP), Atlanta, GA, USA; 3 Maniilaq Association, Kotzebue, AK, USA

**Keywords:** infectious diseases, water, water access

## Abstract

**Background:**

Kivalina is a northwest Alaska barrier island village of 400 people vulnerable to storm surges exacerbated recently by delayed winter sea and shore ice formation. The village has no in-home piped water or sewage; the “washeteria” is the only structure providing public showers, laundry facilities and flush toilets. In October 2004, a storm damaged the washeteria septic system resulting in prolonged facility closures. We assessed rates of gastrointestinal, respiratory and skin infections potentially impacted by prolonged washeteria closures.

**Methods:**

We obtained washeteria closure dates from 2003 to July 2009 and defined >7 day closure as prolonged. We received de-identified data on all Kivalina clinic visits from 2003 to 2009 and selected visits with ICD-9 diagnosis codes for respiratory, skin, or gastrointestinal infection; subsequent same patient/same illness-category visits within 14 days were excluded. We compared annual visit rates, for all ages combined, before (2003–2004) and after (2005–2009) the “2004” storm.

**Results:**

The washeteria had prolonged closures for 34 days (4.7%) in the 2 years 2003–2004 and 864 days (51.7%) between January 2005 and July 2009. Closures ranged from 8 to 248 days. Respiratory infection rates declined significantly from 1.32 visits/person/year in the 2003–2004 period to 0.99 visits/person/year in the 2005–2009 period. There was a significant increase in skin infection rates after 2004, peaking at 0.28 visits/person/year in 2007 and then declining significantly to 0.15 visits/person/year in 2009. Gastrointestinal infection rates remained stable and low throughout (average: 0.05 visits/person/year). No temporal association was observed between respiratory, gastrointestinal or skin infection rates and prolonged washeteria closures.

**Conclusion:**

The Kivalina washeteria was closed frequently and for extended periods between 2005 and 2009. Initial closures possibly resulted in increased skin infection rates. No increase in respiratory or gastrointestinal infections was noted. Evaluation of community adaptations to closures and other factors (e.g. childhood pneumococcal vaccination) would expand understanding of these findings.

## Background

Alaska ranks last in the United States in the provision of in-home piped water. While most villages in Alaska do have in-home piped water, there are many villages that have a water treatment facility from which residents have to collect their own water (referred to as self-haul villages). Recent studies carried out in Alaska comparing rates of disease between piped and self-haul villages have demonstrated an association between lack of in-home piped water and increased rates of hospitalization for respiratory and skin infections (1), presumably due to an insufficient quantity of water available for hand washing, bathing and laundry. Unpublished data indicate that, on average, residents in self-haul villages are accessing about 2 gallons (7.6 litres) per person per day (g/p/d) due to the constraints of having to haul water (personal communication Troy Ritter, ANTHC). The World Health Organization (WHO) recommends a minimum of 13 g/p/d (20 litres) for achieving a basic standard of “low” health concern (2).

Kivalina is an Inupiat village of 400 residents located on a barrier island of the Chukchi sea approximately 80 miles north of Kotzebue. The average elevation is about 10 feet (2 metres). The coast is constantly changing, eroding in some areas and building in others. Sediments are transported from the rivers to the coast, and the shore is carved away or built up by the action of wind, current and waves. However, because of the impacts of climate change, (permafrost thaw, increased storm intensity and delays in winter coastal ice formation), erosion processes are accelerating. The frequency of major storm events is also increasing. Seventy-five percent of the major storm events since 1970 have occurred since 2001 (3). According to the US Army Corps of Engineers, Kivalina is at high risk from erosion hazards. Coastal storms can erode up to 100 feet of shoreline in a single event.

The village has no in-home piped water or sewage; the “washeteria” is the only structure providing public showers, laundry facilities and flush toilets. In October 2004, a storm damaged the washeteria septic system resulting in prolonged facility closures. In 2009, responding to a request by the Kivalina tribal and city governments and Maniilaq Association, the regional tribal health organization, the Alaska Native Tribal Health Consortium (ANTHC) and the Center for Climate and Health undertook a climate change health assessment (4). One outcome of the assessment was a request by the city and tribal council to perform a clinical data review and determine the potential health effects of prolonged washeteria closures resulting from the 2004 storm.

Health care in remote rural Alaskan villages is provided primarily by Community Health Aides (CHAs) supported by medical providers at regional hospitals. CHAs can diagnose and treat common ailments; more complicated cases are referred to the regional hospital. Clinic visits are increasingly entered into the electronic medical record system.

As village residents were still able to access treated water, we targeted our assessment at illnesses that are associated primarily with lack of a sufficient quantity of water exacerbated in this situation by the limited availability of a public facility for bathing and laundry. We assessed rates of Kivalina clinic visits for respiratory and skin infections potentially impacted by prolonged washeteria closures. We also included gastrointestinal (GI) infections for comparison. The 2 main objectives of the analysis were to determine if prolonged closures of the washeteria were followed by increases in rates of clinic visits for GI, respiratory or skin infection and to determine if the annual rates of clinic visits for the same infections changed after the major storm event of 2004.

## Methods

We obtained washeteria closure dates for the period 2003–July 2009 from the Kivalina City offices. The washeteria regularly closes for 1–2 days per week. For this analysis, we defined a prolonged closure as one lasting >7 days. The Kivalina village council requested that electronic records of clinic visits be released to ANTHC from the Maniilaq Health Corporation. We received de-identified data on all Kivalina clinic visits from 2003 to 2009 with the following conditions and related ICD-9 codes that were likely to occur and be diagnosed in the village clinic: GI (codes: 5.9, 7.1, 8.8, 9.1, 787.91), respiratory (codes: 034.0, 460–466, 480–486, 487.1, 490, 786.2) and skin infections (codes: 680–686). Visits by the same person for the same condition within 14 days of an initial visit were considered as a follow-up and thus eliminated from analyses. Prior to 2003, the electronic data were not complete. We used the Alaska State Department of Labor (5) to estimate the Kivalina population between 2003 and 2009; the age breakdown was calculated by using the proportion in age categories for the Northwest Borough.

### Statistical analysis

To assess the effect of the prolonged washeteria closure on disease rates, we used 2 types of statistical models. In the first analysis, we ran a Poisson regression on the weekly number of cases. The explanatory variable in the Poisson regression was the number of days that the washeteria was on a lengthened closure the week prior to (also ran models with a 2-week lag) to the reported disease week rate. The second analysis accounted for the time series nature of the data and had an indicator variable for seasonal adjustment. We ran an autoregression model with a similar structure for the response and explanatory variable except that the response was assumed normal. We tested for the autoregression component of this model using the Durbin-Watson statistic. We tested for heteroscedasticity using the portmanteau Q test statistic (6). We compared annual visit rates, for all ages combined, before (2003–2004) and after (2005–2009) the storm event in 2004 using a 2-sample Poisson test. Analyses were conducted using SAS v 9.2 (Cary, NC) and values <0.05 were considered statistically significant.

## Results

### Washeteria closure

**Fig. 1:**
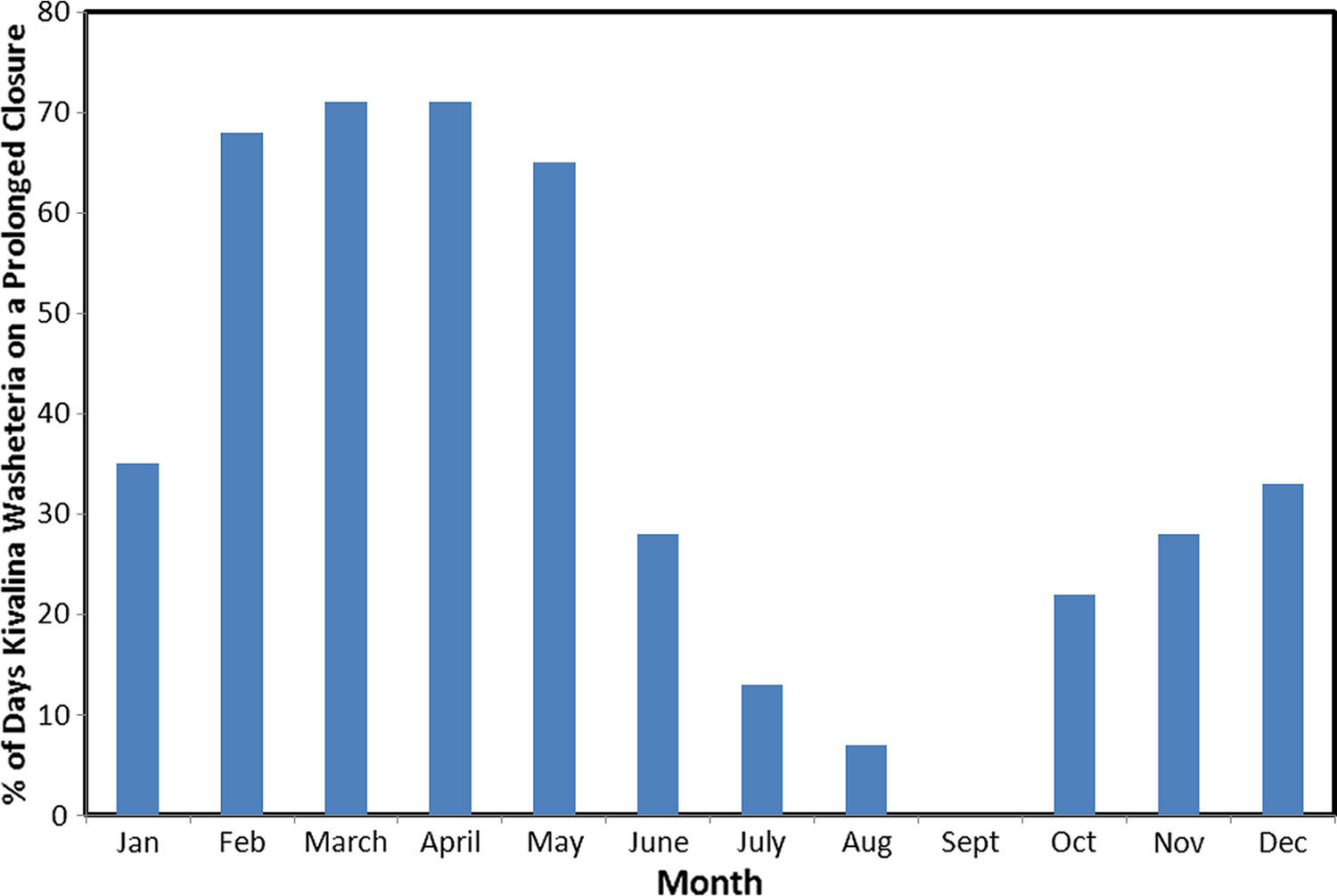
Percentage of days that the washeteria was closed for a prolonged period (>7 days) between 2003 and 2009.

The washeteria in Kivalina regularly closes 1 or 2 days a week. Over the course of the study (2003–2009), the Kivalina washeteria was closed 54% of the days. The routine weekend or 1-day closures made up 17% of the days. The washeteria was closed for prolonged periods for 37% of the days. Multi-month closures (>30 days) first took place in 2005 and occurred every subsequent year. The first recorded damage from a storm surge to the washeteria drain field was in October, 2004. Further damage occurred to the drain field in November, 2004, with the first multi-month closure beginning 30 January, 2005 (Table I). The multi-month closures were usually started or caused by a winter storm. The percentage of days that the washeteria was closed for a prolonged period by month is shown in Fig. 1. Between 2005 and 2009, the washeteria in Kivalina was closed 95% of the days between the months of February and May.

**Table I:** Dates of prolonged (>7 days) closure of the Kivalina washeteria (2003–2009)

Year	% of days closed (prolonged)	Closure blocks	Length of closure
2003	7	Feb 8–17 (9 days)	9 days
		Oct 26–Nov 11	17 days
2004	2	Oct 19–26	8 days
2005	30	Jan 30–May 20	111 days
2006	55	Jan 29–June 4	127 days
		Aug–Oct “compromised”	
		Oct 31–Dec 31	62 days
2007	56	Jan 1–July 5	248 days
		Oct 7–26	20 days
2008	60	Jan 17–May 18	123 days
		May 22–June 4	14 days
		July 9–Aug 13	36 days
		Oct 26–Nov 10	16 days
		Dec 2–31	30 days
2009 (though July)	66	Jan 1–15	45 days
		Feb 18–June 21	124 days

### Disease outcome

**Fig. 2:**
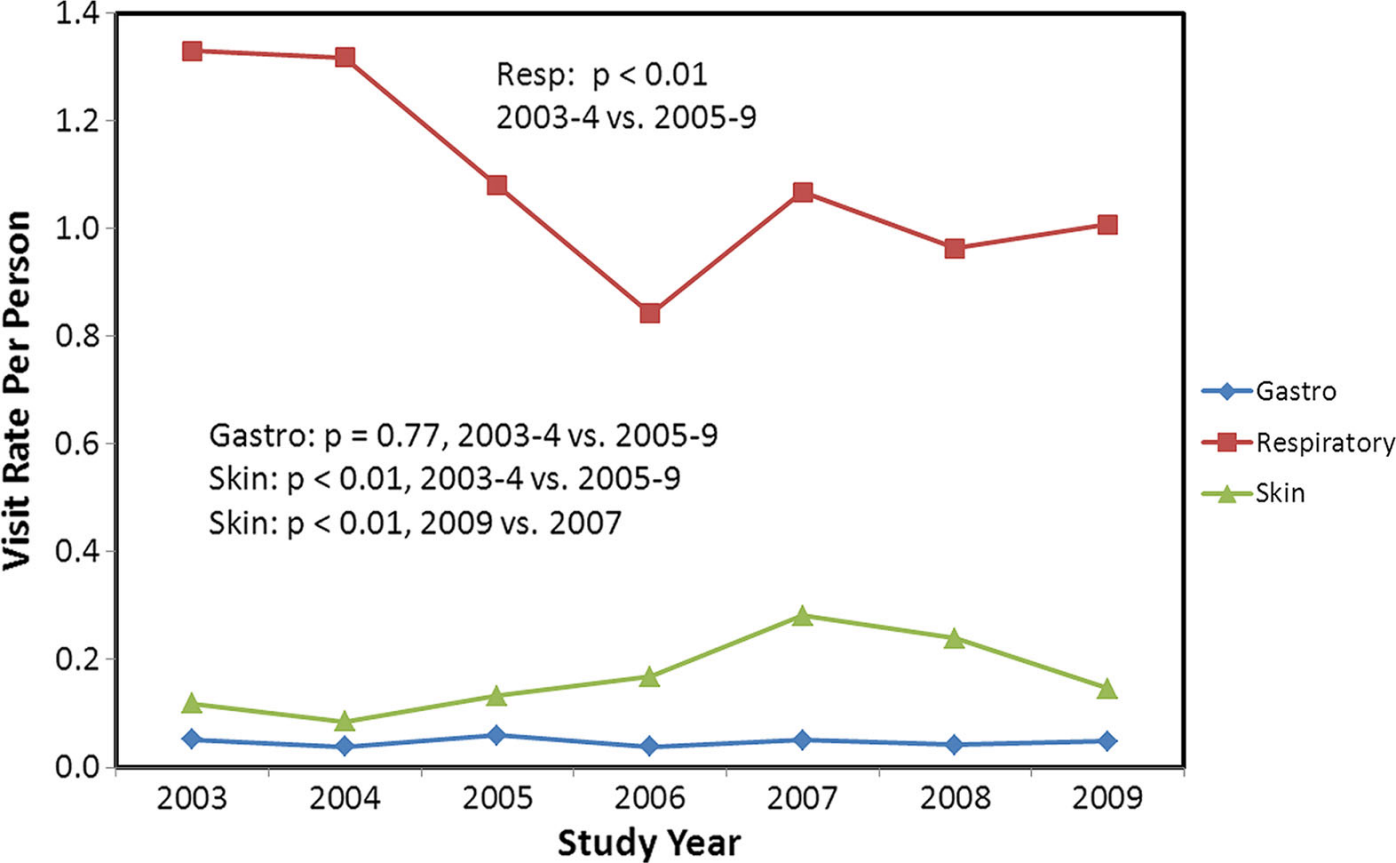
Rates of gastrointestinal, respiratory and skin infection visits per person for the village of Kivalina by study year for all ages combined.

**Fig. 3:**
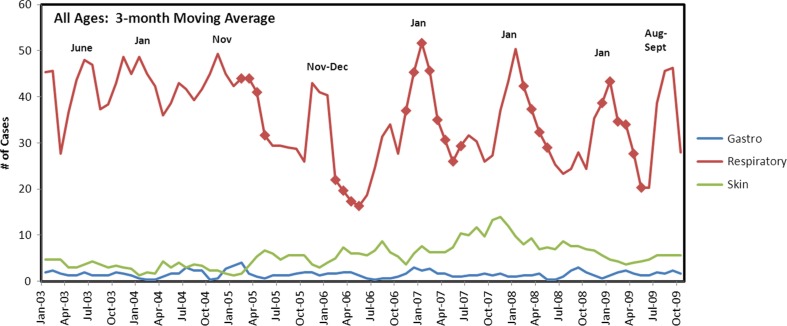
Number of cases of gastrointestinal, respiratory and skin infection visits by year and month at the Kivalina clinic. Respiratory data points from months where washeteria was shut down for the entire month are marked with a diamond marker.

Between 2003 and 2009, the average population size of the village of Kivalina was 395 persons (range 392, 410). There were 3,472 clinic visits for respiratory infections, of which 3,004 (87%) were considered cases (i.e. not for the same person within 14 days of another visit). There were 535 visits for skin infections, of which 465 (87%) were considered cases. For GI illness, there were 145 visits of which 130 (90%) were counted as cases. The illness visit rates by age category for all years demonstrated higher rates at each end of the age spectrum. Respiratory illness visit rates per person per year ranged from a high of 3.7 among children <1 year of age, to a low of 0.5 among those 19–29 years of age and then rising to a rate of 1.0 among those >65 years of age. Skin infection visit rates were <0.5 in all age groups, except 1–4 years old (rate =0.52). GI infection visit rates were <0.1 in all age groups except <1 year old (rate=0.42). Respiratory visit rates were higher than skin or GI and appear to have a seasonal peak during the winter months (Jan–Mar), whereas no such seasonal trend appears evident for GI or skin infection visits (data not shown). The rates by study year for all ages are shown in Fig. 2. The visit rates observed prior to any multi-month closures of the washeteria (2003–2004) are compared to the rates observed in the years that follow (2005–2009). Respiratory illness visit rates declined over the 7 years. The rate of skin infection visits peaked in the winter of 2007–2008 and then declined afterwards (although still higher visit rates than in 2003–2004, *p*=0.03). GI infection visit rates remained stable and low throughout (average: 0.05 visits/person/year). No association was observed between the number of respiratory, GI or skin infection visits and prolonged washeteria closures (Fig. 3).

## Conclusion

This analysis demonstrates a response to a particular request by a community to assess the impact of an environmental event. The Kivalina washeteria was closed frequently and for extended periods after the October 2004 storm. Among the causes evaluated, respiratory infections were the most frequent reason for clinic visit, followed by skin and then GI. Initial closures may have resulted in increased skin infection rates; however, these declined significantly after 2007. There are anecdotal reports that village residents purchased small home washing machines because the washeteria was closed so frequently, perhaps resulting in a decline in skin infection cases. The significant decline in respiratory infections is of interest. This may be partially explained by the increased uptake of paediatric pneumococcal vaccine and possibly less opportunity for people to get infection because they are no longer congregating in the laundry facility. Visits for GI infections remained low throughout this period, and as expected, no change was noted after the storm period as access to treated water was not interrupted. Evaluation of other factors including community adaptive strategies would be valuable.
